# Cell-free DNA promoter hypermethylation in plasma as a diagnostic marker for pancreatic adenocarcinoma

**DOI:** 10.1186/s13148-016-0286-2

**Published:** 2016-11-16

**Authors:** Stine Dam Henriksen, Poul Henning Madsen, Anders Christian Larsen, Martin Berg Johansen, Asbjørn Mohr Drewes, Inge Søkilde Pedersen, Henrik Krarup, Ole Thorlacius-Ussing

**Affiliations:** 1 Department of Gastrointestinal Surgery, Clinical Cancer Research Center, Aalborg University Hospital, Hobrovej 18-22, 9000 Aalborg, Denmark; 2 Department of General Surgery, Hospital of Vendsyssel, Hjørring, Denmark; 3 Department of Clinical Medicine, Aalborg University, Hobrovej 18-22, 9000 Aalborg, Denmark; 4 Section of Molecular Diagnostics, Clinical Biochemistry, Clinical Cancer Research Center, Aalborg University Hospital, Aalborg, Denmark; 5 Unit of Clinical Biostatistics and Bioinformatics, Aalborg University Hospital, Aalborg, Denmark; 6 Mech-Sense, Department of Gastroenterology, Aalborg University Hospital, Aalborg, Denmark

**Keywords:** Pancreatic cancer, Pancreatic adenocarcinoma, Pancreatitis, Diagnostic biomarker, Methylation, Epigenetic, Cell-free DNA

## Abstract

**Background:**

Pancreatic cancer has a 5-year survival rate of only 5–7%. Difficulties in detecting pancreatic cancer at early stages results in the high mortality and substantiates the need for additional diagnostic tools. Surgery is the only curative treatment and unfortunately only possible in localized tumours. A diagnostic biomarker for pancreatic cancer will have a major impact on patient survival by facilitating early detection and the possibility for curative treatment. DNA promoter hypermethylation is a mechanism of early carcinogenesis, which can cause inactivation of tumour suppressor genes. The aim of this study was to examine promoter hypermethylation in a panel of selected genes from cell-free DNA, as a diagnostic marker for pancreatic adenocarcinoma.

**Methods:**

Patients with suspected or biopsy-verified pancreatic cancer were included prospectively and consecutively. Patients with chronic/acute pancreatitis were included as additional benign control groups. Based on an optimized accelerated bisulfite treatment protocol, methylation-specific PCR of a 28 gene panel was performed on plasma samples. A diagnostic prediction model was developed by multivariable logistic regression analysis using backward stepwise elimination.

**Results:**

Patients with pancreatic adenocarcinoma (*n* = 95), chronic pancreatitis (*n* = 97) and acute pancreatitis (*n* = 59) and patients screened, but negative for pancreatic adenocarcinoma (*n* = 27), were included. The difference in mean number of methylated genes in the cancer group (8.41 (95% CI 7.62–9.20)) vs the total control group (4.74 (95% CI 4.40–5.08)) was highly significant (*p* < 0.001). A diagnostic prediction model (age >65, *BMP3*, *RASSF1A*, *BNC1*, *MESTv2*, *TFPI2*, *APC*, *SFRP1* and *SFRP2*) had an area under the curve of 0.86 (sensitivity 76%, specificity 83%). The model performance was independent of cancer stage.

**Conclusions:**

Cell-free DNA promoter hypermethylation has the potential to be a diagnostic marker for pancreatic adenocarcinoma and differentiate between malignant and benign pancreatic disease. This study brings us closer to a clinical useful diagnostic marker for pancreatic cancer, which is urgently needed. External validation is, however, required before the test can be applied in the clinic.

**Trial registration:**

ClinicalTrials.gov, NCT02079363

**Electronic supplementary material:**

The online version of this article (doi:10.1186/s13148-016-0286-2) contains supplementary material, which is available to authorized users.

## Background

Pancreatic cancer is the fourth leading cause of cancer death in the world [1], with a 5-year survival rate of approximately 5–7% [1, 2]. The only curative treatment is complete tumour resection. Unfortunately, only 10–20% of patients receive treatment with the intend to cure. Despite surgery, 50% of patients experience recurrence [3]. Difficulties in detecting the disease at an early stage results in high mortality. This is mainly due to lacking or non-specific symptoms, which are also related to chronic pancreatitis, an essential differential diagnosis and a known risk factor for pancreatic cancer [3, 4]. Often, several complex or invasive techniques such as PET scan (positron emission tomography), CT scan (computed tomography), endoscopic or laparoscopic ultrasound and ERCP (endoscopic retrograde cholangiopancreatography) are needed for the diagnosis and many patients also need a histological evaluation. However, the differentiation between malignant and benign pancreatic disease can be difficult, and even surgery may be needed to establish a definite diagnosis. The only useful biomarker is CA-19-9, which is unspecific as patients with chronic pancreatitis and particularly benign biliary obstruction tend to express high levels of CA-19-9. Moreover, 10% of the population lack the ability to produce CA-19-9, making its utility less apparent [5–7]. It would be a major advance for the patients if a blood-based diagnostic marker was available.

During the development of pancreatic cancer, genetic and epigenetic changes take place. Epigenetic modifications occur at a genomic level, which does not change the DNA sequence. Epigenetic modifications change the DNA conformation and therefore the gene expression. DNA hypermethylation is an epigenetic phenomenon, where a methyl (CH3) residue is added to cytosines preceding guanosines (CpGs) [8–11]. Hypermethylation in the promoter region results in gene silencing, which may be associated with cancer formation [8, 9, 12, 13].

Cancer cells may release cell-free DNA into the blood [14, 15]. DNA hypermethylation can be detected in cell-free DNA in plasma and serum and is potentially tumour specific and useable as blood-based diagnostic markers for pancreatic cancer [14–16].

Thus far, only a few studies with small numbers of patients have evaluated cell-free DNA hypermethylation as a blood-based marker for pancreatic cancer, testing the methylation status of only a single gene or small gene panel [16]. These data have shown a significant difference in DNA hypermethylation between patients with pancreatic cancer and healthy controls [4, 17]. However, the studies had difficulties in differentiating between malignant and benign pancreatic disease [4]. None of the previously examined genes have the potential to serve as an individual diagnostic marker [16]. When developing and testing a biomarker for pancreatic cancer, inclusion of relevant control groups with benign pancreatic disease is very important to enable differentiation of pancreatic cancer-specific hypermethylation and hypermethylation related to pancreatic disease in general [16].

The aim of this study was to test (by methylation-specific polymerase chain reaction (PCR)) cell-free DNA promoter hypermethylation of a panel of 28 genes as a blood-based diagnostic marker for pancreatic adenocarcinoma, including clinical relevant control groups of patients with benign pancreatic disease.

## Methods

### Study design

This study was conducted as a prospective observational cohort study of patients with suspected or biopsy-verified pancreatic cancer admitted to the Department of Gastrointestinal Surgery, Aalborg University Hospital between February 2008 and February 2011 [18]. Additional benign control groups were patients with chronic pancreatitis treated at the hospital or at the outpatient clinic at Aalborg University Hospital between August 2013 and August 2014 and patients admitted with acute pancreatitis at the Department of Gastrointestinal Surgery, Aalborg University Hospital or the Department of General Surgery, Hospital of Vendsyssel between November 2013 and May 2015.

The study was approved by the Research Ethics Committee for the North Denmark Region (N-2013037) and registered in ClinicalTrials.gov (NCT02079363). All participants gave written informed consent.

### Participants

**Fig. 1:**
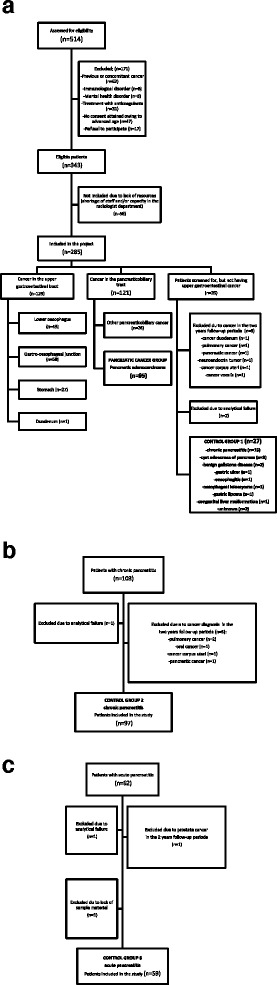
Flow diagram of patients included in the study. **a** Inclusion of patients with pancreatic adenocarcinoma. **b** Inclusion of patients with chronic pancreatitis. **c** Inclusion of patients with acute pancreatitis

Consecutive patients with suspected or biopsy-verified upper gastrointestinal cancer were included prospectively in a study on gastrointestinal cancer and venous thromboembolism [18]. Patients had blood drawn on admission before diagnostic work-up and before any treatment. Patients were divided into the following groups (Fig. 1a). Only patients with pancreatic adenocarcinoma (cancer group) and patients screened, but negative for upper gastrointestinal cancer (control group 1), were included in this study. Patients with chronic pancreatitis (control group 2) had blood drawn during hospitalization or at a scheduled visit in the outpatient clinic. Patients diagnosed with acute pancreatitis (control group 3) were enrolled during the first three days of hospitalization. Patients with chronic pancreatitis and acute pancreatitis were excluded if they had previous cancer history or ongoing anticoagulant therapy.

### Blood sampling and analytical method

Blood samples were obtained by skilled technicians, using peripheral venipuncture from an antebrachial vein, according to the guidelines recommended by the European Concerted Action on Thrombosis [19]. EDTA plasma samples for methylation analysis were centrifuged for 20 min at 4000 rpm at 4 °C and stored within 2 h after sampling in a biobank at −80 °C until methylation analysis.

All methylation analyses were performed by a single expert laboratory scientist.

#### Extraction and deamination

Plasma nucleic acids were extracted using the EasyMag platform (Biomerieux) according to manufacturer’s instruction. Approximately 500-μl EDTA plasma was used for the extraction, and purified nucleic acids were eluted in 35-μl elution buffer (Biomerieux). Five microliters was used for quantitation of extracted DNA, and the remainder was deaminated as previously described by our group [20]. In brief, 30-μl DNA extract was mixed with 60-μl deamination solution and deaminated for 10 min at 90 °C, followed by purification using EasyMag and elution in 25 μl 10 mM KOH [20].

#### First round of PCR amplification

In order to amplify the amount of deaminated DNA of interest, a first round of PCR was conducted using a mix of outer methylation-specific primers (Additional file 1) for all promoter regions tested. The reaction buffer for each sample consisted of 25 μl containing PCR stock, 13 μM MgCl_2_, 0.6 mM dNTP, 250 nM of each outer primer (Additional file 1), 1.5 U Taq polymerase (Bioline) and 0.3 U UNG (Invitrogen). The first round reaction mix was distributed to individual 200-μl PCR tubes and incubated for 5 min at 37 °C (UNG activity), followed by incubation at 95 °C for 5 min and cooling to room temperature. Twenty-five microliters of purified deamination product was added to each tube containing the first round reaction mix. PCR was performed for 20 cycles at 92 °C for 15 s, 55 °C for 30 s and 72 °C for 30 s.

#### Second round of PCR

Ten microliters of mix containing 0.4 μM inner methylation-specific primers and methylation-specific probes (Additional file 1) was distributed in 30 individual wells in a 96-well PCR plate. Ten microliters of first round PCR product was added to 710 μl of reaction mix containing PCR stock, 250 μM dNTP, 10 μM MgCl2, and 15 U Taq polymerase (Bioline). Twenty microliters of the reaction mix was added to each of the 30 wells containing primers and probes. Real-time PCR was carried out for 45 cycles at 94 °C for 15 s, 55 °C for 30 s (annealing and detection) and 72 °C for 30 s.

#### Gene panel

Twenty-eight genes were selected for methylation analysis (Additional file 2). The genes were primarily selected based on a literature review performed by our group prior to this study [16]. The genes selected for the panel had previously been detected as hypermethylated in either cell-free DNA in plasma or serum, pancreatic juice or tumour tissue from patients with pancreatic cancer and in addition unmethylated in samples from healthy individuals. Few additional genes were chosen based on a pilot study on cell-free DNA hypermethylation in colorectal adenocarcinoma (unpublished data).

#### Primer and probe design

All primers and probes were designed using Beacon Designer® (PREMIER Biosoft International, Palo Alto, CA) software and evaluated to be hypermethylation specific by MethPrimer® (The Li Lab, Peking, China) [21]. Primers were designed to be rich in CpGs and to be located up-stream of exon one, which was interpreted as the promoter regions of the genes. The aim was to design PCR products with a length less than 140–150 base pairs, because the cell-free DNA fragments most likely have a length of 160 base pairs consistent with nucleosomal DNA size [22]. The primers and probes were designed and optimized for the present study; however, effort was made to design primers for previously tested promoter sequences (Additional file 1).

Hemi-methylated *MEST* transcript variant 1 was used as reference gene in both the first and second round PCR.

### Outcome

The primary aim was to establish a prediction model for pancreatic adenocarcinoma, enabling differentiation of pancreatic adenocarcinoma patients and a clinical relevant control group of patients screened, but negative for upper gastrointestinal cancer and patients with chronic pancreatitis.

### Statistical methods

Each gene in the gene panel was analysed as a binary variable (hypermethylated or non-methylated).

#### Validation of dichotomous data

We calculated the differences between the threshold cycle (Ct) values of the hemimethylated reference gene *MEST* transcript variant 1 and the Ct values of each gene for which Ct > 0 (∆ Ct). To assess the amount of information lost in the dichotomization, histograms of ∆ Ct for the cancer group and control group 1 combined with control group 2 were produced.

#### Level of cell-free DNA

We calculated the median level (ng/ml) of cell-free DNA for each group. Nonparametric Wilcoxon rank sum test was used for comparison of the cancer group and the benign control groups.

The total number of hypermethylated genes was calculated for each patient. The Kendall’s rank test was used for correlation analysis of total number of hypermethylated genes and level of cell-free DNA.

#### Hypermethylated genes

 The methylation frequency of each gene and the (exact) 95% confidence interval (CI) were calculated for each group. The mean number of hypermethylated genes in each group and the 95% CI was calculated. The means were compared as numerical data with the nonparametric Wilcoxon rank sum test. *p* values less than 0.05 were considered statistically significant.

#### Prediction model development

1. *Screening of each individual variable as a diagnostic marker for pancreatic adenocarcinoma:* Logistic regression was performed separately for each gene in the gene panel and for smoking status, gender and patient age >65. The *p* value and the area under the receiver operating characteristic curve (AUC) were calculated.
2. *The selection of variables:* Variables having a *p* value less than 0.2 were selected for further analysis.
3. *Model selection:* Stepwise backward elimination in logistic regression models was performed to select the relevant variables using 0.05 as the significance level for removal from the model. In the backward elimination algorithm, variables were eliminated one by one to identify the optimal combination of variables representing the highest predictive power. The least significant variable in the variable combination was eliminated in the stepwise procedure. For each intermediate model, the AUC value was calculated.
4. *Determination of the best model:* The decision was based on the model complexity combined with the model performance according to the AUC.
5. *Interactions between the variables*: The significance of interactions between all pairs of variables was assessed in the final model. Interactions with a *p* value less than 0.01 were considered statistically significant.
6. *Validation:* To account for optimism in the internal validation of discriminative model performance (measured by the AUC), “*leave pair out cross validation*” was used [23]. For the calibration performance, Hosmer–Lemeshow test was performed.
7. *Probability score:* For each patient, a probability score was calculated.

All data were analysed using Stata 14.0 software [StataCorp LP, TX].

All authors had full access to the study data and had reviewed and approved the final manuscript.

## Results

Ninety-five patients with confirmed pancreatic adenocarcinoma were included in the study (Fig. 1a). After diagnostic work-up (gastroscopy, endoscopic ultrasound, magnetic resonance (MR) or CT scan), 35 patients without evidence of malignancy were categorized as patients screened but negative for pancreatic adenocarcinoma (control group 1). Eight patients were subsequently excluded from this group (Fig. 1a). Two additional groups of control patients with benign pancreatic disease were included. Overall, 103 patients with chronic pancreatitis (control group 2) and 62 patients with acute pancreatitis (control group 3) were included. Subsequently, six patients from control group 2 and three patients from control group 3 were excluded (Fig. 1b, c). Descriptive data of the four groups are shown in Table 1.

**Table 1:** Descriptive data of the patients

		Pancreatic cancer		Control group 1 (screened negative)		Control group 2 (chronic pancreatitis)		Control group 3 (acute pancreatitis)		Control groups 1 + 2	
*N*		95		27		97		59		124	
Mean age (years) (range)		66	45–85	60	37–82	57	22–87	56	22–87	58	22–87
Sex (% men)		57	60	12	44.44	67	69.07	32	54.24	79	63.71
Smoking status	Currently (%)	30	31.58	11	40.74	64	65.98	23	38.98	75	60.48
	Previously (%)	33	34.74	7	25.93	24	24.74	11	18.64	31	25.00
	Never (%)	30	31.58	9	33.33	9	9.28	23	38.98	18	14.52
	Unknown status (%)	2	2.11	0	0	0	0	2	3.39	0	0
AJCC/UICC staging	I (IA and IB) (%)	11	11.58								
	II (IIA and IIB) (%)	29	30.53								
	III (%)	13	13.68								
	IV (%)	42	44.21								

### Validation of the dichotomous data

There was no clear difference in ∆ Ct between the cancer group and control group 1 combined with control group 2, which indicated that no significant amount of information was lost by dichotomizing the genes as hypermethylated or non-methylated genes regardless of the observed Ct value. Additional file 3a, b lists the distribution of Ct values (0, 0–25, 25–30 and >30) for each gene in within patient group and illustrates a slightly difference in Ct values between the groups, with a tendency towards Ct values in the cancer group being lower compared to the benign control groups. However, due to limited power, the effect of this difference could not be evaluated in the multivariable logistic regression model; consequently, we treated hypermethylation as a dichotomised variable.

### Level of cell-free DNA

Patients with pancreatic adenocarcinoma had a significant higher median level of cell-free DNA (11.60 ng/ml (range 0.60–957.17)) compared to control group 1 with 6.17 ng/ml (range 1.06–48.43), control group 2 with 2.18 ng/ml (0.11–115.44) and 4.09 ng/ml (range 0.65–62.42) for control group 3 (Additional file 4). In addition, the correlation between level of cell-free DNA and number of hypermethylated genes was statistically significant with a Kendall’s *τ* of 0.34 (Additional file 5).

The hypermethylation profile for each patient is illustrated on the heat map plot in Additional file 6. The methylation frequency of each gene is presented in Table 2. The mean number of methylated genes of the whole gene panel (28 genes) was 8.41 (95% CI 7.62–9.20) for the cancer group compared to 4.34 (95% CI 3.85–4.83) for patients with chronic pancreatitis (control group 2), 4.89 (95% CI 4.07–5.71) for patients screened, but negative for pancreatic cancer (control group 1) and 5.34 (95% CI 4.76–5.91) for patients with acute pancreatitis (control group 3). The difference between the cancer group and the three benign control groups was highly statistically significant (Table 3).

**Table 2:** Hypermethylation frequencies for each gene in each group

Gene	Pancreatic cancer (*N* = 95)			Screened negative (*N* = 27)			Chronic pancreatitis (*N* = 97)			Acute pancreatitis (*N* = 59)		
	%	*n*	95% CI	%	*n*	95% CI	%	*n*	95% CI	%	*n*	95% CI
ALX4	17.84	17	(10.78–27.10)	7.41	2	(0.91–24.29)	4.12	4	(1.13–10.22)	1.69	1	(0.04–9.09)
APC	82.11	78	(72.90–89.22)	44.44	12	(25.48–64.67)	54.64	53	(44.21–64.78)	67.80	40	(54.36–79.38)
BMP3	33.68	32	(24.31–44.11)	18.52	5	(6.30–38.08)	3.09	3	(0.64–8.77)	10.17	6	(3.82–20.8)
BNC1	35.79	34	(26.21–46.30)	7.41	2	(0.91–24.29)	5.15	5	(1.69–11.62)	6.78	4	(1.88–16.46)
BRCA1	10.53	10	(5.16–18.51)	14.81	4	(4.19–33.73)	7.22	7	(2.95–14.30)	32.20	19	(20.62–45.64)
CDKN2A	6.32	6	(2.35–13.24)	3.70	1	(0.09–18.97)	2.06	2	(0.25–7.25)	11.86	7	(4.91–22.93)
CDKN2B	12.63	12	(6.70–21.03)	7.41	2	(0.91–24.29)	5.15	5	(1.69–11.62)	11.86	7	(4.91–22.93)
CHFR	1.05	1	(0.03–5.73)	0	0	(0.00–12.77)	3.09	3	(0.64–8.77)	1.69	1	(0.04–9.09)
ESR1	77.89	74	(68.21–85.77)	62.96	17	(42.37–80.60)	60.82	59	(50.39–70.58)	76.27	45	(63.41–86.38)
EYA2	13.68	13	(7.49–22.26)	0	0	(0.00–12.77)	8.25	8	(3.63–15.61)	15.25	9	(7.22–26.99)
GSTP1	3.16	3	(0.66–8.95)	0	0	(0.00–12.77)	1.03	1	(0.03–5.61)	0	0	(0–6.06)
HIC1	15.79	15	(9.12–24.70)	0	0	(0.00–12.77)	6.19	6	(2.30–12.98)	6.78	4	(1.88–16.46)
MESTv2	78.95	75	(69.38–86.64)	44.44	12	(25.48–64.67)	58.76	57	(48.31–68.67)	66.10	39	(52.61–77.92)
MGMT	5.26	5	(1.73–11.86)	0	0	(0.00–12.77)	3.09	3	(0.64–8.77)	0	0	(0–6.06)
MLH1	14.74	14	(8.30–23.49)	22.22	6	(8.62–42.26)	7.22	7	(2.95–14.30)	28.81	17	(17.76–42.07)
NPTX2	74.74	71	(64.78–83.10)	62.96	17	(42.37–80.60)	42.27	41	(32.30–52.72)	49.15	29	(35.89–62.50)
NEUROG1	10.53	10	(5.16–18.51)	11.11	3	(2.35–29.16)	6.19	6	(2.30–12.98)	6.78	4	(1.88–16.46)
RARB	46.32	44	(36.02–56.85)	44.44	12	(25.48–64.67)	28.87	28	(20.11–38.95)	45.76	27	(32.72–59.24)
RASSF1A	42.11	40	(32.04–52.67)	14.81	4	(4.19–33.73)	11.34	11	(5.80–19.39)	16.95	10	(8.44–28.97)
SFRP1	44.21	42	(34.02–54.77)	25.93	7	(11.11–46.28)	17.53	17	(10.55–26.57)	18.64	11	(9.69–30.91)
SFRP2	38.95	37	(29.11–49.50)	18.52	5	(6.30–38.08)	25.77	25	(17.42–35.65)	6.78	4	(1.88–16.46)
SEPT9v2	14.74	14	(8.30–23.49)	0	0	(0.00–12.77)	3.09	3	(0.64–8.77)	1.69	1	(0.04–9.09)
SST	64.21	61	(53.72–73.79)	59.26	16	(38.80–77.61)	30.93	30	(21.93–41.12)	25.42	15	(14.98–38.44)
TFPI2	23.16	22	(15.12–32.94)	3.70	1	(0.09–18.97)	2.06	2	(0.25–7.25)	0	0	(0–6.06)
TAC1	58.95	56	(48.38–68.94)	14.81	4	(4.19–33.73)	35.05	34	(25.64–45.41)	25.42	15	(14.98–38.44)
VIM	3.16	3	(0.66–8.95)	0	0	(0.00–12.77)	0	0	(0–3.73)	0	0	(0–6.06)
WNT5A	8.42	8	(3.71–15.92)	0	0	(0.00–12.77)	1.03	1	(0.03–5.61)	0	0	(0–6.06)
PENK	2.11	2	(0.26–7.40)	0	0	(0.00–12.77)	0	0	(0–3.73)	0	0	(0–6.06)

**Table 3:** Mean number of hypermethylated genes in each group

Group	*N*	Mean number of methylated genes	95% CI	*p* value
Pancreatic cancer	95	8.41	(7.62–9.20)	
Control group 1; screened negative	27	4.89	(4.07–5.71)	
Control group 2; chronic pancreatitis	97	4.34	(3.85–4.83)	
Control group 3; acute pancreatitis	59	5.34	(4.77–5.91)	
Control groups 1 + 2	124	4.46	(4.04–4.88)	<0.0001^a^
Control groups 1 + 2 + 3	183	4.74	(4.40–5.08)	<0.0001^b^

The means were compared as numerical data with nonparametric Wilcoxon rank sum test. *p* values less than 0.05 were considered statistically significant

^a^Significant difference between patients with pancreatic cancer and control groups 1 + 2

^b^Significant difference between patients with pancreatic cancer and control groups 1 + 2 + 3

*CI* confidential interval

### Prediction model development

In the following analyses, we chose to combine control group 1 and 2, as the combined group has symptoms resembling those of pancreatic cancer, which makes a biomarker to distinguish these from pancreatic cancer of utmost clinical relevance. For the remainder of the analysis, patients with acute pancreatitis were excluded because a clinical picture of acute inflammation is rarely seen in pancreatic cancer.

There was a highly significant difference (*p* < 0.001) between the cancer group and control groups 1 + 2 with regard to hypermethylation frequency of ten genes (*APC*, *BMP3*, *BNC1*, *MESTv2*, *NPTX2*, *RASSF1A*, *SFRP1*, *SST*, *TFPI2* and *TAC1*) (Table 4) and significant difference (*p* < 0.05) in seven other genes (*ALX4*, *ESR1*, *HIC1*, *RARB*, *SFRP2*, *SEPT9v2* and *WNT5A*) (Table 4). *VIM* and *PENK* could not be evaluated by logistic regression, as none of the patients in the control group had hypermethylation of these two genes; however, chi-square test found significant difference between the cancer group and the control groups 1 + 2. Despite that, VIM and PENK were excluded from the following analysis because only very few cancer patients had VIM or PENK hypermethylation. (Tables 2 and 4). There was no significant difference in gender; consequently, this variable was excluded from the subsequent analysis. Smoking, however, was a preventive factor for cancer when comparing patients with pancreatic cancer and patients with chronic pancreatitis. Smoking was therefore excluded from the model because it is a known risk factor for cancer. By stratifying the patients into groups according to age (>65 years old, ≤65 years old), a statistically significant difference was found between the cancer group and control groups 1 + 2. Consequently, patient age >65 years old was included as a covariate in the multivariable logistic regression analysis.

**Table 4:** Variables included in the study

	OR	95% CI	*p* value	AUC
*ALX4*	*4.29*	*(1.62; 11.35)*	*0.0034*	*0.57*
*APC*	*4.16*	*(2.21; 7.84)*	*9.67 × 10* ^*−6*^	*0.65*
*BMP3*	*7.37*	*(3.20; 16.95)*	*2.64 × 10* ^*−6*^	*0.64*
*BNC1*	*9.32*	*(3.90; 22.25)*	*5.02 × 10* ^*−7*^	*0.65*
BRCA1	1.21	(0.49; 2.98)	0.6804	0.51
CDKN2A	2.27	(0.66; 11.17)	0.1652	0.52
CDKN2B	2.42	(0.91; 6.40)	0.0757	0.53
CHFR	0.43	(0.04; 4.19)	0.4668	0.51
*ESR1*	*2.23*	*(1.22; 4.07)*	*0.0095*	*0.58*
EYA2	2.30	(0.91; 5.80)	0.0778	0.54
GSTP1	4.01	(0.41; 39.18)	0.2323	0.51
*HIC1*	*3.69*	*(1.37; 9.91)*	*0.0097*	*0.55*
*MESTv2*	*2.99*	*(1.63; 5.49)*	*0.0004*	*0.62*
MGMT	2.24	(0.52; 9.62)	0.2778	0.51
MLH1	1.48	(0.66; 3.31)	0.3448	0.52
*NPTX2*	*3.37*	*(1.88; 6.02)*	*4.34 × 10* ^*−5*^	*0.64*
NEUROG1	1.50	(0.59; 3.86)	0.3969	0.52
*RARB*	*1.81*	*(1.04; 3.15)*	*0.0348*	*0.57*
*RASSF1A*	*5.28*	*(2.69; 10.39)*	*1.4 × 10* ^*−6*^	*0.65*
*SFRP1*	*3.30*	*(1.81; 6.03)*	*0.0001*	*0.62*
*SFRP2*	*2.00*	*(1.12; 3.58)*	*0.0197*	*0.57*
*SEPT9v2*	*6.97*	*(1.94; 25.03)*	*0.0029*	*0.56*
*SST*	*3.04*	*(1.75; 5.30)*	*8.69 × 10* ^*−5*^	*0.64*
*TFPI2*	*12.16*	*(3.51; 42.04)*	*7.96 × 10* ^*−5*^	*0.60*
*TAC1*	*3.25*	*(1.86; 5.69)*	*3.63 × 10* ^*−5*^	*0.64*
*VIM*	–	–	^a^	–
*WNT5A*	*11.31*	*(1.39; 92.08)*	*0.0234*	*0.54*
*PENK*	–	–	^a^	–
Sex	0.85	(0.49; 1.48)	0.5750	0.52
Age 60	3.88	(2.17; 6.92)	4.58 **×** 10^**−**6^	0.66
*Age 65*	*4.14*	*(2.33; 7.33)*	*1.14 × 10* ^*−6*^	*0.67*
Age 70	4.05	(2.04; 8.02)	6.06 **×** 10^**−**5^	0.62

All variables are analysed by simple logistic regression comparing the pancreatic cancer group and control groups 1 + 2

Italic values indicate the genes, where there is significant difference (*p* < 0.05) in hypermethylation frequency between the cancer group and control groups 1 + 2

^**a**^ *VIM* and *PENK* could not be evaluated by logistic regression because none of the patients in the control group had hypermethylation of the two genes; however, chi-square test found significant difference between the cancer group and the control groups 1 + 2. Despite that, *VIM* and *PENK* were excluded from the following analysis because only few cancer patients had *VIM* or *PENK* hypermethylation

Control group 1; patients screened but negative for upper gastrointestinal malignancy

Control group 3; patients with chronic pancreatitis

*OR* odds ratio, *CI* confidential interval, *AUC* area under the receiver operating characteristic curve

**Fig. 2:**
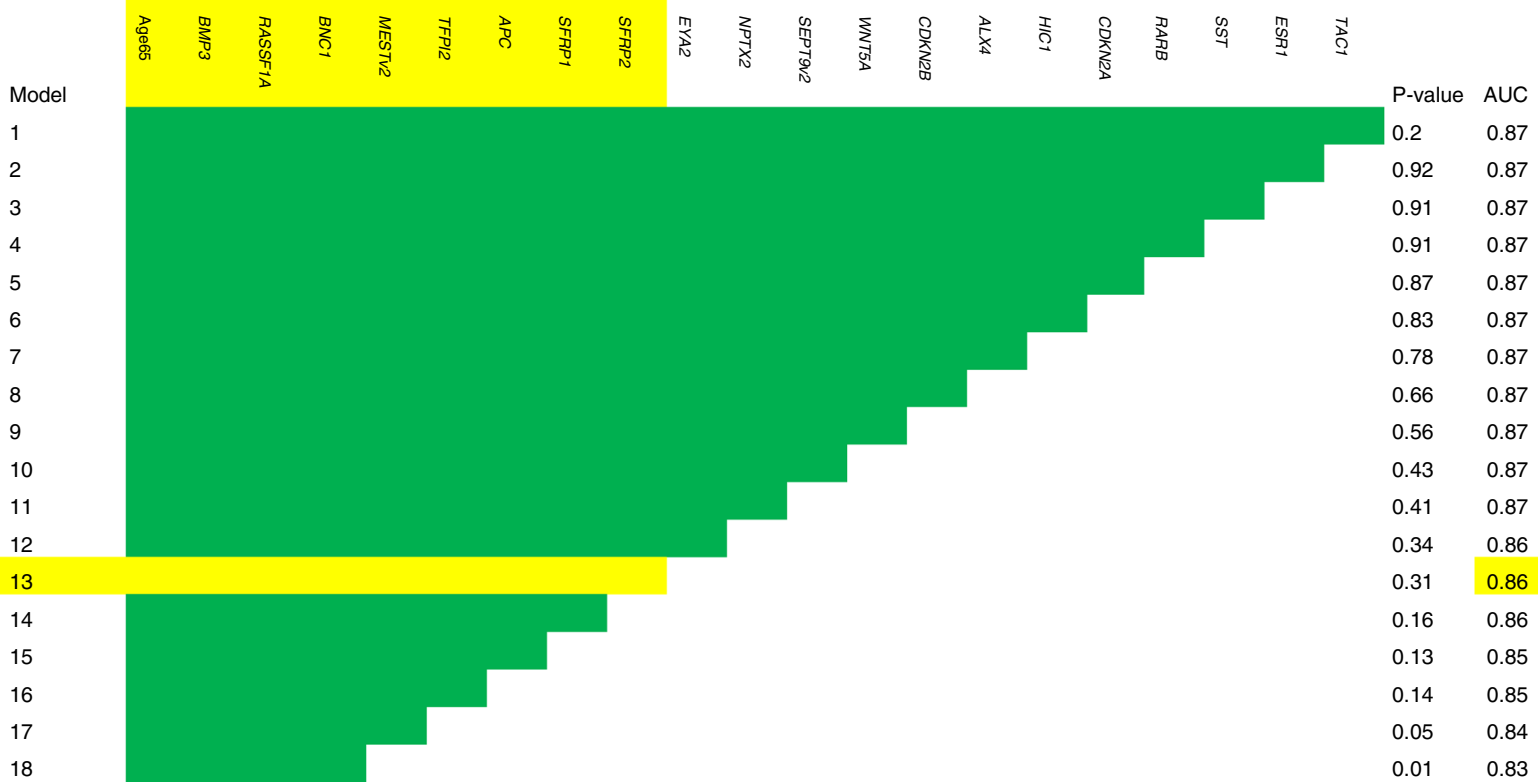
Stepwise selection of genes for the pancreatic cancer diagnostic prediction model. Stepwise selection of genes with the corresponding *p* value and the area under the receiver operating characteristic curve (AUC). Model 13 was determined as the model with the best performance

**Fig. 3:**
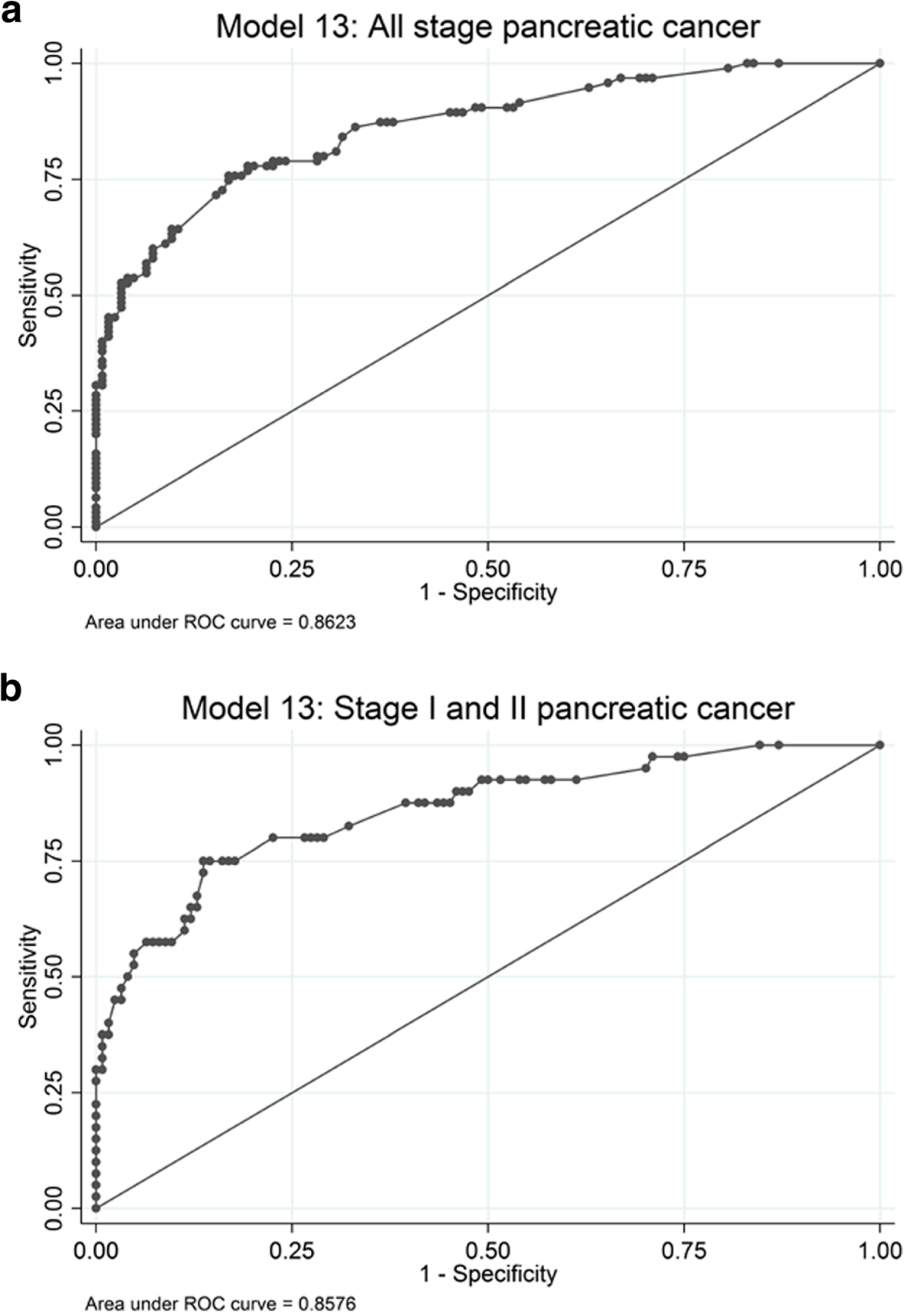
Performance of model 13. **a** Stage I, II, III and IV pancreatic cancer. Model 13 (age >65, *BMP3*, *RASSF1A*, *BNC1*, *MESTv2*, *TFPI2*, *APC*, *SFRP1*, *SFRP2*). AUC = 0.86 (probability cut point of 0.50; sensitivity 76% and specificity 83%).**b** Stage I and II pancreatic cancer. Model 13 (age >65, *BMP3*, *RASSF1A*, *BNC1*, *MESTv2*, *TFPI2*, *APC*, *SFRP1*, *SFRP2*). AUC = 0.86 (probability cut point of 0.50; sensitivity 73% and specificity 83%)

All genes with an individual *p* value below 0.20 (20 genes out of 28 examined genes) and patient age >65 were included in the multivariable logistic regression model. Backward stepwise selection was performed. Figure 2 illustrates the stepwise elimination of variables from the model and the corresponding AUC. The initial model (model 1) with 20 genes had an AUC of 0.87 (Fig. 2). Removing the 12 least significant genes from the model and leaving eight genes (model 13; age >65, *BMP3*, *RASSF1A*, *BNC1*, *MESTv2*, *TFPI2*, *APC*, *SFRP1* and *SFRP2*) resulted in an AUC of 0.86 (95% CI 0.81–0.91) (Figs. 2 and 3a). The mean probability for having pancreatic adenocarcinoma was 0.67 (0.61–0.72) for cancer patients and 0.26 (0.22–0.29) for the control groups 1 + 3. Model 13 was determined as the model with the best performance (probability cut point of 0.50; sensitivity 76% and specificity 83%). There were no statistically significant interactions between variables in model 13. The model was well calibrated (p = 0.40) and had an estimated optimism in AUC of 0.03.

Forty patients had stage I or II tumours. Model 13 had an apparent AUC of 0.86 (95% CI 0.79–0.92) for stage I/II tumours (probability cut point of 0.50; sensitivity 73% and specificity 83%) (Fig. 3b) with an optimism in AUC of 0.06.

## Discussion

We examined cell-free DNA promoter hypermethylation of 28 genes in the plasma of a large cohort of patients with pancreatic adenocarcinoma and compared it to different clinical relevant control groups. We designed the gene-panel primary based on our literature review addressing genes aberrantly methylated in pancreatic adenocarcinoma [16]. This approach was used, to evaluate the overall diagnostic performance of genes which previously had been examined separately as diagnostic markers for pancreatic cancer. The panel was composed of genes previously detected as hypermethylated in plasma/serum and tumour tissue in relation to pancreatic adenocarcinoma (*BNC1* [17, 24, 25], *NPTX2* [4, 24–26], *PENK* [4, 14, 25], *CDKN2A* [4, 26, 27], *RASSF1A* [4, 24, 27], *SFRP1* (*SARP2*) [4, 25], *APC* [24, 27], *BRCA1* [28, 29], *CDKN2B* [28, 30], *ESR1* [25, 28], *MGMT* [24, 28], *MLH1* [28, 31] and *RARB* [28, 32]), genes earlier found to be hypermethylated in pancreatic juice or tumour tissue from patients with pancreatic adenocarcinoma (*BMP3* [24], *EYA2* [24], *GSTP1* [29], *HIC1* [25, 33], *SFRP2* [24], *TFPI2* [25], *VIM* [25], *NEUROG1* [24, 25], *TAC1* [24, 25], *CHFR* [24] and *WNT5a* [24, 25]) and genes found based on a pilot study on cell-free DNA hypermethylation in colorectal adenocarcinoma (*ALX4*, *MESTv2*, *SEPT9v2* and *SST*). To our knowledge, this is the first study to examine cell-free DNA hypermethylation in a wide selection of genes by methylation-specific PCR in a large group of patients with either malignant or benign pancreatic disease.

A statistically significant difference in the hypermethylation status in 19 out of the 28 genes was found when comparing pancreatic adenocarcinoma patients and a control group containing patients screened, but negative for pancreatic cancer, as well as in patients with chronic pancreatitis. Cell-free DNA hypermethylation of *BMP3*, *MESTv2*, *SST*, *TFPI2*, *TAC1*, *ALX4*, *HIC1*, *SFRP2*, *SEPT9v2* and *WNT5A* has not previously been described in the literature in relation to pancreatic cancer. Yi et al. described *BNC1* hypermethylation to have a sensitivity of 79% and a specificity of 89% when comparing pancreatic cancer and healthy individuals [17]. We found BNC1 to be hypermethylated in only 36% of pancreatic cancer patients with a specificity of 94%. Park et al. examined hypermethylation of a small gene panel (*NPTX2*, *RASSF1A*, *SFRP1*, *UCHL1*, *PENK* and *CDKN2A*) by methylation-specific PCR [4]. The gene panel could differentiate pancreatic cancer from healthy controls; however, it was not able to discriminate benign and malignant pancreatic disease.

Our study shows that cell-free DNA hypermethylation is detectable in both malignant and benign pancreatic disease. However, patients with pancreatic adenocarcinoma have a higher level of hypermethylated genes in plasma-derived cell-free DNA. Consistent with previous studies, our gene panel did not demonstrate a single gene, which could be used as an individual diagnostic marker for pancreatic cancer. This result suggests that a larger gene panel is needed to achieve sufficient accuracy [16]. We developed a diagnostic prediction model (age >65, *BMP3*, *RASSF1A*, *BNC1*, *MESTv2*, *TFPI2*, *APC*, *SFRP1* and *SFRP2*), which was able to differentiate between pancreatic adenocarcinoma and a large control group of great clinical relevance. The control group included patients with chronic pancreatitis or patients referred to the hospital with symptoms of pancreatic cancer. The AUC was high, and the predictive value of our model is superior to the predictive value of CA-19-9, which currently is the only blood-based biomarker for pancreatic cancer. Particularly keeping in mind that CA-19-9 is highly dependent on the Lewis blood group status of the patients. Only Le^a+b−^ or Le^a-b+^ individuals are able to express CA-19-9 but not Le^a−b−^ individuals, which represent 5–10% of the Caucasian population [34]. In a recent study, CA-19-9 could differentiate patients with stage I–II pancreatic cancer from patients with chronic pancreatitis with an AUC of 0.77 (sensitivity of 53% and a specificity of 92%) and pancreatic cancer patients from patients with benign biliary obstruction with an AUC of only 0.65 [5]. Our study included patients with stage I–IV pancreatic adenocarcinoma. It is most important to diagnose patients with stage I and II disease as early detection at this stage of the disease has the potential to improve the outcome of surgery. We tested our model on stage I and II disease and found an AUC of 0.86. This finding shows that the performance of the prediction model is independent of the cancer stage. DNA hypermethylation is detectable in plasma even in an early stage of the disease and thereby potentially usable as an early blood-based diagnostic marker.

In order to further differentiate DNA hypermethylation related to malignant and benign pancreatic disease, patients with acute pancreatitis were included. The aim was to achieve a more basic understanding of hypermethylated DNA during the course of an acute pancreatic inflammatory reaction, which has not been described earlier in literature. Our study shows that DNA hypermethylation takes place during pathological conditions in the pancreas including acute inflammation. However, the changes are more pronounced in patients with pancreatic adenocarcinoma.

### Limitations

Our study has some limitations. The study was exploratory, showing training data only, which is known to produce an overestimation of the test performance due to overfitting. Validation of the results in an independent cohort is needed to substantiate the results.

Patients were not matched according to age, which one should be aware of because epigenetic changes can be a part of ageing [35]. To address this problem, we incorporated age as a covariate in our prediction model.

In addition, comparison of the performance of our prediction model to CA-19-9 would have been relevant. Unfortunately, this was impossible, as CA-19-9 was not available on two thirds of the patients as this test was first implemented in 2010 at our department.

The difference in sensitivity of the genes analysed in our study and the sensitivity of genes examined in previous studies by others might be due to the use of different primer sequences. Several methods are described for methylation analysis which furthermore makes inter-study comparison difficult [36].

We performed methylation-specific PCR, which is a quantitative method using hemimethylated *MEST* transcript variant 1 as a reference gene [20]. However, our study lacked sufficient power to conduct a quantitative analysis. Therefore, we analysed hypermethylation as a binary variable, which unfortunately results in loss of the quantitative information.

At the end of the analyses, we discovered that the use of UNG (Invitrogen) had a tendency to lower the sensitivity compared to the use of COD UNG (ArcticZymes). All our samples are analysed using UNG (Invitrogen) because it was not possible to repeat all analyses with COD UNG (ArcticZymes) due to the lack of sample material.

### Strengths

However, the study also has several strengths. We tested cell-free DNA hypermethylation of a broad gene panel in the plasma from a large group of patients with pancreatic adenocarcinoma, all included prospectively and consecutively, before the diagnostic workup and before any treatment.

In addition, we included a large utmost relevant group of control patients with either benign pancreatic disease or with symptoms mimicking pancreatic cancer, which all are patients clinically hard to differentiate from patients with pancreatic cancer.

We performed methylation-specific PCR based on an optimized bisulfite treatment protocol [20]. This method has several advantages, due to a high recovery from samples with minute amounts of DNA (<0.01 ng/ml) and a rapid deamination of DNA (less than 2 h) [20].

We developed a diagnostic prediction model for pancreatic adenocarcinoma with a high performance, independent of cancer stage. In addition, the diagnostic prediction model only had a modest optimism in performance by intern validation.

Diagnostic biomarkers for pancreatic cancer are lacking. We developed a diagnostic test, which has the great advantage of being blood based and thereby minimally invasive. In general, blood-based markers are of great benefit to the patients compared to tissue-based markers, as the latter entail a risk of complications. Furthermore, due to the deep location of the pancreas in the upper abdomen, biopsies may be difficult to obtain, why blood-based markers are of utmost importance regarding pancreatic disease.

## Conclusions

Our study demonstrates statistically significant differences in cell-free DNA hypermethylation of several genes between malignant and benign pancreatic diseases. Patients with pancreatic adenocarcinoma have a highly significant number of hypermethylated genes compared to patients with benign pancreatic diseases. A panel of hypermethylated genes (*BMP3*, *RASSF1A*, *BNC1*, *MESTv2*, *TFPI2*, *APC*, *SFRP1* and *SFRP2*) are able to differentiate between patients with pancreatic adenocarcinoma and a most relevant control group. Based on our study, alterations in cell-free DNA hypermethylation have the potential of serving as blood-based biomarkers for the diagnosis of pancreatic adenocarcinoma. External validation is however required before the biomarker can be applied in daily clinical practice.

## Additional files

Additional file 1: DNA sequences for probes and primers. (DOCX 24 kb)
Additional file 2: Characteristics of genes used in the gene panel. (DOCX 23 kb)
Additional file 3: a: Distribution of Ct values in the cancer group and control group 1. b: Distribution of Ct values in the control group 2 and control group 3. (ZIP 26 kb)
Additional file 4: Level of cell-free DNA. (DOCX 15 kb)
Additional file 5: Correlation between level of cell-free DNA (ng/ml) and total number of hypermethylated genes. (DOCX 15 kb)
Additional file 6: Heat map of hypermethylated genes in each patient. (XLSX 38 kb)
