# Supplementary material for: Cell-free DNA promoter hypermethylation in plasma as a diagnostic marker for pancreatic adenocarcinoma
Source: Clin Epigenetics. 2016 Nov 16;8:117. doi: 10.1186/s13148-016-0286-2 (PMC5112622; doi:10.1186/s13148-016-0286-2)
Supplement: Additional file 1: — DNA sequences for probes and primers. (DOCX 24 kb) [file 13148_2016_286_MOESM1_ESM.docx]

| **Additional file 1. DNA sequences for probes and primers** | | | | |
| --- | --- | --- | --- | --- |
| Gene |  | DNA sequence | Position | Amplicon size |
| TAC1 M1 | NC_000007.14 | ATC GTA AGG TAT TGA GTA GGC | 97732062 to 97732082 |  |
| TAC1 M2 |  | TCT CGA TAA CTA CCG CCG | 97732109 to 97732126 | 64 |
| TAC1 M beacon |  | (HEX)CGA TCG ATC C+GA AC+G C+GC TCT CGA TCG(Dabcyl) | 97732086 to 97732100 |  |
| TAC1 Am |  | TAA GGA GGT TGG GAT AAA TAT C | 97732043 to 97732064 |  |
| TAC1 Bm |  | TCT CGA TAA CTA CCG CCG | 97732109 to 97732126 | 83 |
| SST M1 | NC_000003.12 | GCG TCG AGA TGT TGT TTT GTC | 187670279 to 187670299 |  |
| SST M2 |  | CCA AAA CCA AAA CGA TAA ACA ACG | 187670234 to 187670257 | 65 |
| SST M beacon |  | (HEX)CGA TCG ACC AAC +GC+G CAC TAA CGA TCG(Dabcyl) | 187670260 to 187670274 |  |
| SST Am |  | TAG TTC GGT TTT CGC GGC GTC | 187670260 to 187670274 |  |
| SST Bm |  | CCA AAA CCA AAA CGA TAA ACA ACG | 187670234 to 187670257 | 81 |
| APC M1 | NC_000005.10 | AGT GCG GGT CGG GAA GC | 112737732 to 112737748 |  |
| APC M2 |  | AAT CGA CGA ACT CCC GAC G | 112737805 to 112737823 | 91 |
| APC M beacon |  | (HEX)CGC GAT CGT TG+G ATG +CG+G AAT CGC G(Dabcyl) | 112737773 to 112737785 |  |
| APC Am |  | ATT GCG GAG TGC GGG TC | 112737725 to 112737741 |  |
| APC Bm |  | AAT CGA CGA ACT CCC GAC G | 112737805 to 112737823 | 98 |
| MLH1 M1 | NC_000003.12 | TGG TTT TTT GGC GTT AAA ATG TC | 36993529 to 36993552 |  |
| MLH1 M2 |  | AAA TAA CTT CCC CCG CCG | 36993606 to 36993623 | 94 |
| MLH1 M beacon |  | (HEX)CGC GAT CTC +GTC CAA CC+G CC+G AAT ATC GCG(Dabcyl) | 36993569 to 36993592 |  |
| MLH1 Am |  | TGG TTT TTT GGC GTT AAA ATG TC | 36993529 to 36993552 |  |
| MLH1 Bm |  | CAT CTC TTT AAT AAC ATT AAC TAA CCG | 36993626 to 36993652 | 123 |
| SFRP1 M1 | NC_000008.11 | GGA GTT GAT TGG TTG CGC | 41309508 to 41309525 |  |
| SFRP1 M2 |  | CGC GAC ACT AAC TCC G | 41309435 to 41309450 | 90 |
| SFRP1 M beacon |  | (HEX)CGC GAT G+GT T+CG +GTC G+TA ATC GCG(Dabcyl) | 41309482 to 41309493 |  |
| SFRP1 Am |  | GAG GCG ATT GGT TTT CGC | 41309567 to 41309584 |  |
| SFRP1 Bm |  | CGC GAC ACT AAC TCC G | 41309435 to 41309450 | 149 |
| CHFR M1 | NC_000012.12 | GTT TCG GTT TTA GTT TCG TAT TTC | 132887175 to 132887198 |  |
| CHFR M2 |  | CGA CTC CTA CGT CTA AAC GCG | 132887257 to 132887277 | 102 |
| CHFR M beacon |  | (HEX)CGC GAT CCG +CA+C GT+C CAT CGC G(Dabcyl) | 132887235 to 132887244 |  |
| CHFR Am |  | GTT TCG GTT TTA GTT TCG TAT TTC | 132887175 to 132887198 |  |
| CHFR Bm |  | CCC TAA AAA CGA CTC CTA CG | 132887267 to 132887286 | 111 |
| RASSF1A M1 | NC_000003.12 | GGG AGG CGT TGA AGT C | 50340882 to 50340897 |  |
| RASSF1A M2 |  | GTA CTT CGC TAA CTT TAA ACG | 50340821 to 50340841 | 76 |
| RASSF1A M beacon |  | (HEX)CGC GAT TCG +TT+C G+GT TCG CTC GCG(Dabcyl) | 50340846 to 50340859 |  |
| RASSF1A Am |  | GGG AGG CGT TGA AGT C | 50340882 to 50340897 |  |
| RASSF1A Bm |  | A ATA AAC TCA AAC TCC CCC G | 50340782 to 50340801 | 115 |
| CDKN2A M1 | NC_000009.12 | TTT CGA GTA TTC GTT TAT AGC | 21975019 to 21975036 |  |
| CDKN2A M2 |  | TTT CTT CCT CCG ATA CTA ACG | 21974925 to 21974945 | 111 |
| CDKN2A M beacon |  | (HEX)CGA CGT G+AA +AGA +TAT CG+C G+GT ACG TCG(Dabcyl) | 21974988 to 21975002 |  |
| CDKN2A Am |  | TGT TCG GAG TTA ATA GTA TTT TTT TC | 21975033 to 21975058 |  |
| CDKN2A Bm |  | TTT CTT CCT CCG ATA CTA ACG | 21974925 to 21974945 | 133 |
| RARB M1 | NC_000003.12 | GGG TAT CGT CGG GGT AGA TTC | 25428402 to 25428423 |  |
| RARB M2 |  | TCG ACC AAT CCA ACC GAA ACG | 25428495 to 25428515 | 113 |
| RARB M beacon |  | (HEX)CGC GAC GAA +TA+C GTT +CCG AAT CGC G(Dabcyl) | 25428421 to 25428435 |  |
| RARB Am |  | AGT AGG GTT TGT TTG GGT ATC | 25428388 to 25428408 |  |
| RARB Bm |  | TCG ACC AAT CCA ACC GAA ACG | 25428495 to 25428515 | 127 |
| ESR1 M1 | NC_000006.12 | GGG ATT GTA TTT GTT TTC GTC | 151807705 to 151807725 |  |
| ESR1 M2 |  | ACG CAA CGC ATA TCC CG | 151807793 to 151807809 | 104 |
| ESR1 M beacon |  | (HEX)CGC GAT GAA +CGA +CCC G+AC GAT CGC G(Dabcyl) | 151807722 to 151807735 |  |
| ESR1 Am |  | GTT TTG GGA TTG TAT TTG TTT TC | 151807700 to 151807722 |  |
| ESR1 Bm |  | ACG CAA CGC ATA TCC CG | 151807793 to 151807809 | 109 |
| BRCA1 M1 | NC_000017.11 | TCG TGG TAA CGG AAA AGC GCG | 43125409 to 43125429 |  |
| BRCA1 M2 |  | CCG TCC AAA AAA TCT CAA CG | 43125346 to 43125365 | 83 |
| BRCA1 M beacon |  | (HEX)CGA TCG G+CG GCG +TG+A GCG ATC G(Dabcyl) | 43125362 to 43125371 |  |
| BRCA1 Am |  | GT TTT TTG GTT TTC GTG GTA AC | 43125420 to 43125441 |  |
| BRCA1 Bm |  | AAA CCC CAC AAC CTA TCC CCC G | 43125327 to 43125348 | 114 |
| MESTv2 M1 | NC_000007.14 | CGA CGT TTT AGT TTC GAG TC | 130486250 to 130486269 |  |
| MESTv2 M2 |  | CGC TTC CTA AAA CCA AAA ATT CTC G | 130486312 to 130486336 | 86 |
| MESTv2 M beacon |  | (HEX)CGA TCG G+TG +GT+C G+GG TTC GAT CG(Dabcyl) | 130486278 to 130486289 |  |
| MESTv2 Am |  | GCG ATG GGT TTG TGC GC | 130486225 to 130486242 |  |
| MESTv2 Bm |  | GAA AAA CCG ATT ACG CAT ACG | 130486337 to 130486355 | 130 |
| MGMT M1 | NC_000010.11 | GAT ATG TTG GGA TAG TTC GC | 129467213 to 129467232 |  |
| MGMT M2 |  | GCA CTC TTC CGA AAA CGA AAC G | 129467311 to 129467332 | 119 |
| MGMT M beacon |  | (HEX)CGC GAT CG+T ATC G+TT +TG+C GAT +TTA TCG CG(Dabcyl) | 129467279 to 129467294 |  |
| MGMT Am |  | GAT ATG TTG GGA TAG TTC GC | 129467213 to 129467232 |  |
| MGMT Bm |  | AAA AAA CTC CGC ACT TCC G | 129467322 to 129467342 | 129 |
| SEPT9v2 M1 | NC_000017.11 | GTT TAG TAT TTA TTT TCG AAG TTC | 77373542 to 77373560 |  |
| SEPT9v2 M2 |  | CCT CCG CGC GAC CCG | 77373467 to 77373481 | 91 |
| SEPT9v2 M beacon |  | (FAM)CGA CGT ATT TAG TTG CGC GTT GAT CGA CGT CG(Dabcyl) | 77373511 to 77373530 |  |
| SEPT9v2 Am |  | GTT TAG TAT TTA TTT TCG AAG TTC | 77373542 to 77373560 |  |
| SEPT9v2 Bm |  | GCC GAA AAC GCT TCC TCG | 77373442 to 77373459 | 118 |
| VIM M1 | NC_000010.11 | ATA TTT ATC GCG TTT TCG TTC | 17229337 to 17229357 |  |
| VIM M2 |  | ACG AAC CTA ATA AAC ATA ACT ACG | 17229416 to 17229439 | 102 |
| VIM M beacon |  | (FAM)CGA CGT GTT CGC GTT ATC GTC GTC GAC GTC G(Dabcyl) | 17229377 to 17229395 |  |
| VIM Am |  | GAG GTT TTC GCG TTA GAG AC | 17229296 to 17229315 |  |
| VIM Bm |  | ACG AAC CTA ATA AAC ATA ACT ACG | 17229416 to 17229439 | 143 |
| EYA2 M1 | NC_000020.11 | CGG AGG TAG CGG TAA C | 46894866 to 46894881 |  |
| EYA2 M2 |  | CGA TAC GAA CGA ACG AAC G | 46894941 to 46894959 | 93 |
| EYA2 M beacon |  | (FAM)CGC GAT TTC GGT TTC GTC GGA TTC GTA TCG CG(Dabcyl) | 46894914 to 46894933 |  |
| EYA2 Am |  | AGG AGG CGG AGG TAG C | 46894860 to 46894875 |  |
| EYA2 Bm |  | CGA CGC GAT ACG AAC G | 46894949 to 46894964 | 104 |
| BMP3 M1 | NC_000004.12 | AGT GGA GAC GGC GTT C | 81031024 to 81031039 |  |
| BMP3 M2 |  | CTT ACT ACG CTA ACC CAA CG | 81031101 to 81031120 | 96 |
| BMP3 M beacon |  | (FAM)CGT CGA GCG GGT GAG GTT CGC GTA TCG ACG(Dabcyl) | 81031052 to 81031069 |  |
| BMP3 Am |  | TAG CGT TGG AGT GGA GAC | 81031015 to 81031032 |  |
| BMP3 Bm |  | CCA ACC CCA CTT ACT ACG | 81031112 to 81031129 | 114 |
| ALX4 M1 | NC_000011.10 | TTT TTC GGA GGC GAT AAG TTC | 44309934 to 44309954 |  |
| ALX4 M2 |  | CGA ACC CGA CTC TTA ACG | 44309869 to 44309886 | 85 |
| ALX4 M beacon |  | (FAM)CGC GAT TGT CGG TCG TCG TTA AAG TAT CGC G(Dabcyl) | 44309902 to 44309920 |  |
| ALX4 Am |  | GTC GGG AGG GTT CGT C | 44309968 to 44309983 |  |
| ALX4 Bm |  | CGA ACC CGA CTC TTA ACG | 44309869 to 44309886 | 114 |
| SFRP2 M1 | NC_000004.12 | GTT TTT CGG AGT TGC GCG C | 153789028 to 153789046 |  |
| SFRP2 M2 |  | CCG AAA AAC TAA CAA CCG ACG | 153788948 to 153788968 | 98 |
| SFRP2 M beacon |  | (HEX)CGA CGT TTG TAG CGT TTC GTT CGC GTT GTT ACG TCG(Dabcyl) | 153789000 to 153789023 |  |
| SFRP2 Am |  | GTT TTT CGG AGT TGC GC GC | 153789028 to 153789046 |  |
| SFRP2 Bm |  | CTC TTC GCT AAA TAC GAC TCG | 153788922 to 153788942 | 124 |
| NEUROG1 M1 | NC_000005.10 | GTT GAT TTG ATC GTC GGC | 135535925 to 135535942 |  |
| NEUROG1 M2 |  | CTC GCC TAC AAA AAC CAC G | 135535879 to 135535897 | 63 |
| NEUROG1 M beacon |  | (HEX)CGC GAT GCC C+GA CC+G ATC TCC TAA ATC GCG(Dabcyl) | 135535899 to 135535916 |  |
| NEUROG1 Am |  | GTT TAT ACG AGT TGA TTT GAT C | 135535931 to 135535952 |  |
| NEUROG1 Bm |  | CTT AAC CTA ACC TCC TCG | 135535860 to 135535882 | 92 |
| NTPX2 M1 | NC_000007.14 | AGG TTA GAG TGT CGA GTA GC | 98617280 to 98617299 |  |
| NTPX2 M2 |  | TCG AAA ATC GCG TAC ACC G | 98617342 to 98617360 | 80 |
| NTPX2 M beacon |  | (HEX)CGC GAT CGG TG+C GGT TGT GAG A+CG GTG ATC GCG(Dabcyl) | 98617306 to 98617322 |  |
| NTPX2 Am |  | TTC GGT AGG TTA GAG TGT C | 98617274 to 98617291 |  |
| NTPX2 Bm |  | CTA TCG TCT CGA AAA TCG CG | 98617349 to 98617368 | 94 |
| TFPI2 M1 | NC_000007.14 | TAT TTT TTA GGT TTC GTT TCG GC | 93890809 to 93890831 |  |
| TFPI2 M2 |  | AAA CGA CCC GAA TAC CCG | 93890759 to 93890776 | 72 |
| TFPI2 M beacon |  | (HEX)CGC GAT CGT CGG T+CG GA+C GTT CGT TGA TCG CG(Dabcyl) | 93890787 to 93890804 |  |
| TFPI2 Am |  | TAT TTT TTA GGT TTC GTT TCG GC | 93890809 to 93890831 |  |
| TFPI2 Bm |  | CGA CTT TCT ACT CCA AAC G | 93890745 to 93890763 | 86 |
| BNC1 M1 | NC_000015.10 | GTA GGT AGT TAG TTG GTT TTC | 83284403 to 83284423 |  |
| BNC1 M2 |  | GAA ACA AAC GAC CCG AAA CG | 83284467 to 83284486 | 83 |
| BNC1 M beacon |  | (FAM)CGC GAT CGT ATT TA+C GGG AGT +CGG AGT TTG ATC GCG(Dabcyl) | 83284440 to 83284461 |  |
| BNC1 Am |  | GTA GGT AGT TAG TTG GTT TTC | 83284403 to 83284423 |  |
| BNC1 Bm |  | GCG AAA ATT CTC TAT ACG | 83284491 to 83284505 | 102 |
| CDKN2B M1 | NC_000009.12 | TAT TGT ACG GGG TTT TAA GTC | 22009107 to 22009127 |  |
| CDKN2B M2 |  | TTC CCT TCT TTC CCA CG | 22009019 to 22009035 | 108 |
| CDKN2B M beacon |  | (HEX)CGC GAT CGA +CGA +CGG GAG GGT AAT GGA TCG CG(Dabcyl) | 22009082 to 22009099 |  |
| CDKN2B Am |  | GGT CGT TCG GTT ATT GTA C | 22009120 to 22009138 |  |
| CDKN2B Bm |  | TTC CCT TCT TTC CCA CG | 22009019 to 22009035 | 119 |
| WNT5A M1 | NC_000003.12 | CGT GGA ATA GTT GTT TGC | 55487294 to 55487311 |  |
| WNT5A M2 |  | TTA AAA CAA AAC TAA AAT ACG | 55487177 to 55487197 | 134 |
| WNT5A M beacon |  | (HEX)CGC GAT CAA CCT AAT C+GA AAC +GCA ACT AAA GAT CGC G(Dabcyl) | 55487247 to 55487269 |  |
| WNT5A Am |  | CGT GGA ATA GTT GTT TGC | 55487294 to 55487311 |  |
| WNT5A Bm |  | CGA ACC TAA ACT CCC G | 55487159 to 55487174 | 152 |
| PENK M1 | NC_000008.11 | AGG CGA TTT GAG TCG TTT TTA C | 56446123 to 56446144 |  |
| PENK M2 |  | GAC AAC CTC AAC AAA AAA TCG | 56446032 to 56446052 | 112 |
| PENK M beacon |  | (HEX)CGC GAT CAA AGT TGT +CGG T+CG GGA GG ATC GCG(Dabcyl) | 56446096 to 56446113 |  |
| PENK Am |  | CGC GTT ATT TCG GGA ATC | 56446148 to 56446165 |  |
| PENK Bm |  | GAC AAC CTC AAC AAA AAA TCG | 56446032 to 56446052 | 133 |
| H1C1 M1 | NC_000017.11 | TTC GGT TTT CGC GTT TTG TTC | 2056080 to 2056100 |  |
| H1C1M2 |  | CGA AAA CTA TCA ACC CTC G | 2056153 to 2056171 | 91 |
| H1C1 M beacon |  | (FAM)CGC GAC GGT CGT CGT TCG GGT TCG CG (Dabcyl) | 2056131 to 2056146 |  |
| H1C1 Am |  | GAT ATA ACG TTT TTT TCG CGT C | 2056054 to 2056075 |  |
| H1C1 Bm |  | ATA CCC GCC CTA ACG CCG | 2056179 to 2056196 | 142 |
| GSTP1 M1 | NC_000011.10 | TCG GGG TGT AGC GGT C | 67583673 to 67583688 |  |
| GSTP1 M2 |  | CCC AAT ACT AAA TCA CGA CG | 67583741 to 67583760 | 87 |
| GSTP1 M beacon |  | (HEX)CGCGAT GTC G+G+C GGG AGT TCG ATC GCG (Dabcyl) | 67583701 to 67583715 |  |
| GSTP1 Am |  | AGG GCG TTT TTT TGC GGT C | 67583649 to 67583667 |  |
| GSTP1 Bm |  | CCC AAT ACT AAA TCA CGA CG | 67583741 to 67583760 | 111 |
| *MESTv1 M1 | NC_000007.14 | CGC GGT AAT TAG TAT ATT TC | 130492085 to 130492107 |  |
| *MESTv1 M2 |  | GCT ACG ACA CTA CGC TTA CG | 130492135 to 130492159 | 74 |
| *MESTv1 M beacon |  | (HEX)CGC GAT CGG +TA+G T+TG +CGT TAT CGC G(Dabcyl) | 130492121 to 130492133 |  |
| *MESTv1 U1 |  | TGT TGT GGT AAT TAG TAT ATT TT | 130492088 to 130492107 |  |
| *MESTv1 U2 |  | CAA CCA CTC CAA CAT ACA CTA CA | 130492154 to 130492171 | 83 |
| *MESTv1 U beacon |  | (FAM)CGC GAG +TA+G T+TG +TG+T TT+T GTT CGC G(Dabcyl) | 130492123 to 130492137 |  |
| **MESTv1 A |  | GGT TTT AAA AGT T/CGG TGT TTA TT | 130492052 to 130492074 |  |
| **MEST1v1 B |  | CCI AAC AAC TAC AAC CAC TCC | 130492162 to 130492182 | 130 |
| a, * Hemimethylated reference gene MEST transcript variant 1  b, ** Un-methylated primer for the reference gene MEST transcript variant 1  M1; methylation specific forward primer for the array (inner primer)  M2; methylation specific reverse primer for the array (inner primer)  M beacon; methylation specific probe  Am; methylation specific forward primer for the nested/semi-nested PCR (outer primer/ 1. Round of PCR)  Bm; methylation specific reverse primer for the nested/semi-nested PCR (outer primer/ 1. Round of PCR) | | | | |
