# Supplementary material for: Cell-free DNA promoter hypermethylation in plasma as a diagnostic marker for pancreatic adenocarcinoma
Source: Clin Epigenetics. 2016 Nov 16;8:117. doi: 10.1186/s13148-016-0286-2 (PMC5112622; doi:10.1186/s13148-016-0286-2)
Supplement: Additional file 2: — Characteristics of genes used in the gene panel. (DOCX 23 kb) [file 13148_2016_286_MOESM2_ESM.docx]

| Additional file 2. Characteristics of genes used in the gene-panel | | |
| --- | --- | --- |
| *Gene* | *Name* | *Mechanism of action* |
| *ALX4* | Aristaless-Like Homeobox 4 | - Expressed in the mesenchymal cells of developing bones, limbs, hair, teeth, and mammary tissue. - ALX4-deficient mice exhibit body wal defects. - ALX4 mutations result in defects in skull ossification - Regulates acitivites of LEF-1 and important component in the Wnt/β-catenin pathway - May be involved in the epithelial to mesenchymal transition in cancer. |
| *APC* | Adenomatous Polyposis Coli | - Encodes a tumor suppressor protein that acts as an antagonist of the Wnt-pathway. - It is also involved in other processes including cell migration and adhesion, transcriptional activation, and apoptosis. - May be involved in increased angiogenesis in cancer. - Defects in the gene causes familial adenomatous polyposis coli (FAP). - Disease mutations tend to be clustered in a small region designated the mutation cluster region (MCR) and result in a truncated protein product. |
| *BMP3* | Bone Morphogenetic Protein 3 | - Belongs to the transforming growth factor-beta superfamily. - The protein induces bone formation - BMP4/9 is involved in angiogenesis in mice. |
| *BNC1* | Basonuclein 1 | - Encodes a protein present in the basal cell layer of the epidermis and in hair follicles. - Found in abundance in germ cells of testis and ovaries. - Regulates in keratinocyte proliferation. - Regulator for rRNA transcription. |
| *BRCA1* | Breast cancer 1 | - Encodes a nuclear phosphoprotein that plays a role in maintaining genomic stability. - Acts as tumor suppressor. - Combines with other tumor suppressors, DNA damage sensors, and signal transducers to form a large multi-subunit protein complex (BRCA1-associated genome surveillance complex), which is associated with RNA polymerase II and interacts with histone deacetylase complexes. - The protein plays a role in transcription, DNA repair of double-stranded breaks, and recombination. |
| *CDKN2B* | Cyclin-dependent kinase inhibitor 2B (P15) | - The gene lies adjacent to the tumor suppressor gene CDKN2A (P16) in a region that is frequently mutated and deleted in a wide variety of tumors. - Encodes a cyclin-dependent kinase inhibitor, which forms a complex with CDK4 or CDK6, and prevents the activation of the CDK kinases. - The protein functions as a cell growth regulator that controls cell cycle G1 progression. - The expression of this gene is induced by TGF beta |
| *CHFR* | Checkpoint with forkhead and ring finger domains | - Encodes an E3 ubiquitin-protein ligase. - The ubiquitin-protein is involved in the antephase checkpoint that regulates cell cycle entry into mitosis. - May play a key role in cell cycle progression and tumorigenesis. |
| *ESR1* | Estrogen Receptor 1 | - Encodes an estrogen receptor involved in DNA binding and activation of transcription. - The protein localizes to the nucleus where it may form a homo-/heterodimer with estrogen receptor 2. - Estrogen receptors are involved in breast cancer, endometrial cancer, and osteoporosis. - Cardiomyocyte Estrogen Receptor Alpha increases antiogenesis lymphangiogenesis and reduces fibrosis in female mice hearts. - Estrogen Receptor Beta increases intratumeral tumor blood vessel growth and decreases expression of VEGF. |
| *EYA2* | Transcriptional coactivator and phosphatase2 | - Encodes a member of the eyes absent (EYA) family of proteins. - The protein may play a role in eye development. - May act as a transcriptional activator. |
| *GSTP1* | Glutathione S-transferase pi 1 | - Glutathione S-transferases (GSTs) are a family of enzymes that play an important role in detoxification. - GSTP1 proteins are thought to play a role in susceptibility to cancer, and other diseases. |
| *HIC1* | Hypermethylated In Cancer 1 | - Encodes a transcriptional repressor. - May be involved in the development of head, neck, face, limbs and ventral body wall. - HIC1 is involved in the transcriptional repression of FGF-BP1 by TGF-β signaling regulation of angiogenesis in cancer. - Hypermethylation or deletion has been associated with different tumours. |
| *MGMT* | O-6-Methylguanine-DNA Methyltransferase | - Encodes an enzyme involved in DNA-repair. - The enzyme removes alkylating lesions at the O6 of the guanine residues, transferring the O6 alkyl-group to a cysteine residue on the enzyme. - This can undermine cancer therapy using alkylating agents, and thus inhibitors of MGMT activity are of interest in cancer research. - Cells lacking MGMT expression have induced angiogenic expression. |
| *MLH1* | MutL Homolog 1 | - Encodes a protein involved in the DNA mismatch repair system. - As part of a multiplex enzyme, it introduces single-strand breaks near the mismatch and thus generates new entry points for the exonuclease EXO1 to degrade the strand containing the mismatch. - It is also involved in DNA damage signalling, a process which induces cell cycle arrest and can lead to apoptosis in case of major DNA damages. |
| *NEUROG1* | Neurogenin 1 | - Encodes a transcriptional regulator, involved in neuronal differentiation. - Diseases associated with NEUROG1 include Moebius Syndrome and Medulloblastoma. - It is involved in the regulation of the Wnt-mediated beta catenin signalling and target gene transcription. |
| *NPTX2* | Neural Pentraxin II | - Encodes a member of the neuronal pentraxins. - The protein is related to synapses and related to C-reactive protein. - Plays a role in the clustering of the AMPA-type glutamate receptors at established synapses, resulting in non-apoptotic cell death of dopaminergic nerve cells (tumour suppressor????). - The protein is upregulated in Parkinsons disease. |
| *p16* | Cyclin-dependent kinase inhibitor 2A | - Encodes 3 alternatively spliced distinct proteins, 2 of which encode structurally related isoforms known to function as inhibitors of CDK4 kinase. - The remaining transcript is an ARF product, which functions as a stabilizer of the tumour suppressor protein p53 as it can interact with, and sequester, the E3 ubiquitin-protein ligase MDM2, a protein responsible for the degradation of p53. - This gene is frequently mutated or deleted in a wide variety of tumours, and is known to be an important tumor suppressor. |
| *PENK* | Proenkephalin | - Encodes proenkephalin associated with the signalling by GPCR and NF-kappaB family pathway. - Met- and Leu-enkephalins compete with and mimic the effects of opiates. - Plays a role in pain perception and responses to stress. |
| *RARB* | Retinoic Acid Receptor Beta | - Encodes a member of the thyroid-steroid hormone receptor superfamily of nuclear transcriptional regulators. - This receptor localizes to the cytoplasm and to subnuclear compartments. It binds retinoic acid, which mediates cellular signalling in embryonic morphogenesis, cell growth and differentiation. - It is thought that this protein limits growth of many cell types by regulating gene expression. - The gene was first identified in a hepatocellular carcinoma where it flanks a hepatitis B virus integration site. Alternate promoter splicing result in multiple in multiple transcript variants. |
| *RASSF1A* | Ras Association Domain Family Member 1 | - Encodes a protein similar to the RAS effector proteins. - Loss or altered expression of this gene has been associated with the pathogenesis of a variety of cancers, which suggests the tumour suppressor function of this gene. - The inactivation of this gene was found to be correlated with the hypermethylation of its CpG-island promoter region. - The encoded protein was found to interact DNA repair protein XPA. - The protein was also shown to inhibit the accumulation of cyclin D1, and thus induce cell cycle arrest. - Required for death receptor-dependent apoptosis. |
| *SEPT9* | Septin 9 | - Encodes a member of the septin family. - Involved in cytokinesis and cell cycle control. - It is a candidate ovarian tumour suppressor gene. - Mutations in this gene cause hereditary neuralgic amyotrophy, also known as neuritis with brachial predilection. - A chromosomal translocation involving this gene on chromosome 17 and the MLL gene on chromosome 11 results in acute myelomonocytic leukaemia. - Involved in cell division, movement and development of new blood vessels. |
| *SFRP1* | Secreted Frizzled-Related Protein 1 | - Encodes a member of the SFRP family that contains a cysteine-rich domain homologous to the putative Wnt-binding site of Frizzled proteins. - Members of this family act as soluble modulators of Wnt signalling; epigenetic silencing of SFRP genes leads to deregulated activation of the Wnt-pathway which is associated with cancer. - Fuses with wif-1 to inhibit various factors in the Wnt pathway including angiogenesis. |
| *SFRP2* | Secreted Frizzled-Related Protein 2 | - Encodes a member of the SFRP family that contains a cysteine-rich domain homologous to the putative Wnt-binding site of Frizzled proteins. - SFRP’s act as soluble modulators of Wnt signalling. Methylation of this gene is a potential marker for the presence of colorectal cancer. - SFRP2 is thought to up-regulate angiogenesis in a mouse-model via a calcineurin/NFAT pathway. |
| *SST* | Somatostatin | - The hormone somatostatin has active 14aa and 28aa forms that are produced by alternate cleavage of the single preprotein encoded by this gene. - Somatostatin is expressed throughout the body and inhibits the release of numerous secondary hormones by binding to high-affinity G-protein-coupled somatostatin receptors. - This hormone is an important regulator of the endocrine system through its interactions with pituitary growth hormone, thyroid stimulating hormone, and most hormones of the gastrointestinal tract. - Somatostatin also affects rates of neurotransmission in the central nervous system and proliferation of both normal and tumorigenic cells. |
| *TAC1* | Tachykinin, Precursor 1 (substance p) | - Encodes four products of the tachykinin peptide family, substance P and neurokinin A, as well as the related peptides, neuropeptide K and neuropeptide gamma. - These hormones are thought to work as neurotransmitters which interact with nerve receptors and smooth muscle cells. - They are known to induce behavioural responses and function as vasodilators and secretagogues. - Substance P is an antimicrobial peptide with antibacterial and antifungal properties. |
| *TFPI2* | Tissue Factor Pathway Inhibitor 2 | - Encodes a member of the kunitz-type serine proteinase inhibitor family. - The protein can inhibit a variety of serine proteases including factor VIIa/tissue factor, factor Xa, plasmin, trypsin, chymotrypsin and plasma kallikrein. - This gene has been identified as a tumour suppressor gene in several types of cancer. - VEGF induce TFPI2 suggesting this as a negative feedback loop, thereby making TFPI2 an inhibitor of angiogenesis. |
| *VIM* | Vimentin | - Encodes a member of the intermediate filament family. - Intermediate filaments, along with microtubules and actin microfilaments, make up the cytoskeleton. - Maintains cell shape, integrity of the cytoplasm, and stabilizes cytoskeletal interactions. - Involved in the immune response and controls the transport of the low-density lipoprotein (LDL) derived cholesterol from a lysosome to the site of esterification. - It functions as an organizer of a number of critical proteins involved in attachment, migration, and cell signalling. - Mutations in this gene causes a dominant, pulverulent cataract. |
| *WNT5A* | Wingless-Type MMTV Integration Site Family, Member 5A | - Encodes a member of the WNT pathway. - The protein is implicated in oncogenesis and in several developmental processes, including regulation of cell fate and patterning during embryogenesis. |
| *MESgT1 (UM)* | Mesoderm Specific Transcript | - Encodes a member of the alpha/beta hydrolase superfamily. - It is imprinted, exhibiting preferential expression from the parental allele in fetal tissue, and isoform-specific imprinting in lymphocytes. - The loss of imprinting of this gene has been linked to certain types of cancer and may be due to promoter switching. |
| *MEST1 (M)* |  |  |
| Gene functions are cross-matched with the ref-seq database on www.ncbi.gov | | |
