# Supplementary material for: Cell-free DNA promoter hypermethylation in plasma as a diagnostic marker for pancreatic adenocarcinoma
Source: Clin Epigenetics. 2016 Nov 16;8:117. doi: 10.1186/s13148-016-0286-2 (PMC5112622; doi:10.1186/s13148-016-0286-2)
Supplement: Additional file 3: — a: Distribution of Ct values in the cancer group and control group 1. b: Distribution of Ct values in the control group 2 and control group 3. (ZIP 26 kb) [file 13148_2016_286_MOESM3_ESM.zip › Additional file 3 a.docx]

| **Additional file 3 a. Distribution of Ct- values** | | | | | | | | | | | | | | | | |
| --- | --- | --- | --- | --- | --- | --- | --- | --- | --- | --- | --- | --- | --- | --- | --- | --- |
| Gene | Pancreatic cancer (N=95) | | | | | | | | Control group 1 (screened negative) (N=27) | | | | | | | |
|  | Ct = 0 | | Ct 0-25 | | Ct 25-30 | | Ct > 30 | | Ct = 0 | | Ct 0-25 | | Ct 25-30 | | Ct > 30 | |
|  | n | % | n | % | n | % | n | % | n | % | n | % | n | % | n | % |
| *ALX4* | 78 | 82 | 1 | 1 | 4 | 4 | 12 | 13 | 25 | 93 | 0 | 0 | 0 | 0 | 2 | 7 |
| *APC* | 17 | 18 | 24 | 25 | 40 | 42 | 14 | 15 | 15 | 56 | 1 | 4 | 6 | 22 | 5 | 19 |
| *BMP3* | 63 | 66 | 11 | 12 | 13 | 14 | 8 | 8 | 22 | 81 | 0 | 0 | 1 | 4 | 4 | 15 |
| *BNC1* | 61 | 64 | 1 | 1 | 7 | 7 | 26 | 27 | 25 | 93 | 0 | 0 | 0 | 0 | 2 | 7 |
| *BRCA1* | 85 | 89 | 1 | 1 | 3 | 3 | 6 | 6 | 23 | 85 | 0 | 0 | 0 | 0 | 4 | 15 |
| *P16* | 89 | 94 | 1 | 1 | 0 | 0 | 5 | 5 | 26 | 96 | 0 | 0 | 0 | 0 | 1 | 4 |
| *CDKN2B* | 83 | 87 | 0 | 0 | 5 | 5 | 7 | 7 | 25 | 93 | 0 | 0 | 0 | 0 | 2 | 7 |
| *CHFR* | 94 | 99 | 0 | 0 | 1 | 1 | 0 | 0 | 27 | 100 | 0 | 0 | 0 | 0 | 0 | 0 |
| *ESR1* | 21 | 22 | 53 | 56 | 21 | 22 | 0 | 0 | 10 | 37 | 5 | 19 | 11 | 41 | 1 | 4 |
| *EYA2* | 82 | 86 | 1 | 1 | 3 | 3 | 9 | 9 | 27 | 100 | 0 | 0 | 0 | 0 | 0 | 0 |
| *GSTP1* | 92 | 97 | 1 | 1 | 0 | 0 | 2 | 2 | 27 | 100 | 0 | 0 | 0 | 0 | 0 | 0 |
| *HIC1* | 80 | 84 | 1 | 1 | 5 | 5 | 9 | 9 | 27 | 100 | 0 | 0 | 0 | 0 | 0 | 0 |
| *MESTv2* | 20 | 21 | 20 | 21 | 43 | 45 | 12 | 13 | 15 | 56 | 3 | 11 | 6 | 22 | 3 | 11 |
| *MGMT* | 90 | 95 | 0 | 0 | 3 | 3 | 2 | 2 | 27 | 100 | 0 | 0 | 0 | 0 | 0 | 0 |
| *MLH1* | 81 | 85 | 1 | 1 | 4 | 4 | 9 | 9 | 21 | 78 | 0 | 0 | 2 | 7 | 4 | 15 |
| *NPTX2* | 24 | 25 | 24 | 25 | 31 | 33 | 16 | 17 | 10 | 37 | 1 | 4 | 8 | 30 | 8 | 30 |
| *NEUROG1* | 85 | 89 | 1 | 1 | 4 | 4 | 5 | 5 | 24 | 89 | 0 | 0 | 1 | 4 | 2 | 7 |
| *RARB* | 51 | 54 | 0 | 0 | 9 | 9 | 35 | 37 | 15 | 56 | 0 | 0 | 3 | 11 | 9 | 33 |
| *RASSF1A* | 55 | 58 | 0 | 0 | 0 | 0 | 40 | 42 | 23 | 85 | 0 | 0 | 0 | 0 | 4 | 15 |
| *SFRP1* | 53 | 56 | 3 | 3 | 13 | 14 | 26 | 27 | 20 | 74 | 0 | 0 | 0 | 0 | 7 | 26 |
| *SFRP2* | 58 | 61 | 19 | 20 | 6 | 6 | 12 | 13 | 22 | 81 | 0 | 0 | 1 | 4 | 4 | 15 |
| *SEPT9v2* | 81 | 85 | 2 | 2 | 3 | 3 | 9 | 9 | 27 | 100 | 0 | 0 | 0 | 0 | 0 | 0 |
| *SST* | 34 | 36 | 29 | 31 | 26 | 27 | 6 | 6 | 11 | 41 | 1 | 4 | 14 | 52 | 1 | 4 |
| *TFPI2* | 73 | 77 | 3 | 3 | 14 | 15 | 5 | 5 | 26 | 96 | 0 | 0 | 1 | 4 | 0 | 0 |
| *TAC1* | 39 | 41 | 1 | 1 | 8 | 8 | 47 | 49 | 23 | 85 | 0 | 0 | 0 | 0 | 4 | 15 |
| *VIM* | 92 | 97 | 0 | 0 | 1 | 1 | 2 | 2 | 27 | 100 | 0 | 0 | 0 | 0 | 0 | 0 |
| *WNT5A* | 87 | 92 | 0 | 0 | 2 | 2 | 6 | 6 | 27 | 100 | 0 | 0 | 0 | 0 | 0 | 0 |
| *PENK* | 93 | 98 | 0 | 0 | 0 | 0 | 2 | 2 | 27 | 100 | 0 | 0 | 0 | 0 | 0 | 0 |
| Threshold cycle value; Ct value.  N; Total number of patients within the group.  n; number of patients with a specific Ct-value | | | | | | | | | | | | | | | | |
