# Supplementary material for: Cell-free DNA promoter hypermethylation in plasma as a diagnostic marker for pancreatic adenocarcinoma
Source: Clin Epigenetics. 2016 Nov 16;8:117. doi: 10.1186/s13148-016-0286-2 (PMC5112622; doi:10.1186/s13148-016-0286-2)
Supplement: Additional file 4: — Level of cell-free DNA. (DOCX 15 kb) [file 13148_2016_286_MOESM4_ESM.docx]

| **Additional file 4. Level of cell-free DNA** | | | | |
| --- | --- | --- | --- | --- |
|  | N | Median (ng/ml) | Range | P-value |
| C. pancreas | 87 | 11.6 | 0.60-957.17 |  |
| Screened negative | 27 | 6.17 | 1.06-48.43 | 0.006 |
| Chronic pancreatitis | 87 | 2.18 | 0.11-115.44 | <0.001 |
| Acute pancreatitis | 40 | 4.09 | 0.65-62.42 | <0.001 |
| Missing | 37 | - | - | - |
| We calculated the median level of cell-free DNA in each group. Wilcoxon rank sum test was used to compare the cancer group to each of the benign groups. | | | | |

Level of cell-free DNA illustrated by box-plots of each patient group.

Control group 1; patients screened, but negative for upper gastrointestinal cancer.

Control group 2; patients with chronic pancreatitis.

Control group 3; patients with acute pancreatitis.
