# Supplementary material for: Cell-free DNA promoter hypermethylation in plasma as a diagnostic marker for pancreatic adenocarcinoma
Source: Clin Epigenetics. 2016 Nov 16;8:117. doi: 10.1186/s13148-016-0286-2 (PMC5112622; doi:10.1186/s13148-016-0286-2)
Supplement: Additional file 5: — Correlation between level of cell-free DNA (ng/ml) and total number of hypermethylated genes. (DOCX 15 kb) [file 13148_2016_286_MOESM5_ESM.docx]

**Additional file 5. Correlation between level of cell-free DNA (ng/ml) and total number of hypermethylated genes.**

The correlation between level of cell-free DNA (ng/ml) and total number of hypermethylated genes was analyzed using Kendall’s rank correlation test resulting in a Kendall’s τ of 0.34
